# Evaluation of video review tools for assessing non-technical skills in emergency department resuscitation teams: a systematic review

**DOI:** 10.1186/s12873-023-00895-7

**Published:** 2023-11-29

**Authors:** Emily G Alexander, Fraser Denny, Malcom WG Gordon, Cieran McKiernan, David J Lowe

**Affiliations:** 1 University of Glasgow, Glasgow, UK; 2 Queen Elizabeth University Hospital, Glasgow, UK; 3 Institute of Health and Wellbeing, University of Glasgow, Glasgow, UK

**Keywords:** Non-technical skills, Emergency department, Resuscitation team, Trauma, Video review, T-NOTECHS

## Abstract

**Background and importance:**

Use of video review in medicine is established in contexts such as surgery. Although not widely used in the emergency department (ED), some centres use it to evaluate non-technical skills (NTS) to support teaching and quality improvement.

**Objective:**

There is no consensus on assessment of NTS using video review in the ED and the purpose of this review was to identify tools used in this context.

**Design, setting and participants:**

Studies were identified using Embase, Medline, CINAHL and Google Scholar. Inclusion criterion for the review was NTS of resuscitation teams working within the ED were assessed using video review. A systematic search method was used, and results were synthesised after search criteria was checked by two independent reviewers. Authors settled on the same 9 studies eligible for inclusion.

**Outcome measures and analysis:**

Reliability and validity of tools identified for use in this context. Due to the heterogeneity of studies, no meta-analysis occurred.

**Main results:**

There are 9 studies included in the review. The review was registered with PROSPERO (Ref No: CRD42022306129). Four unique tools were identified – 6 studies used T-NOTECHS, 1 used TTCA-24, 1 used CALM and 1 used the Communication tool. T-NOTECHS is validated in the literature for use in this context.

**Conclusion:**

T-NOTECHS is the tool of choice for assessing ED teams in this context.

**Supplementary Information:**

The online version contains supplementary material available at 10.1186/s12873-023-00895-7.

## Introduction

Providing high quality resuscitation to patients presenting in the emergency department requires a coordinated performance of interventions to achieve resuscitation success and patient survival; [1] this requires non-technical skills (NTS). [2] NTS include skills such as leadership, communication, situational awareness, decision making and teamwork. [3] Leadership skills are correlated with increased quality of CPR and the International Liaison Committee on Resuscitation recommends that “specific teamwork training” should be taught on courses. [4] The importance of evaluating NTS within teams is increasing, as are the number of tools used to assess them. [1] Early examples are adapted from the aviation industry, where measuring NTS was already commonplace. [5, 6].

Assessing NTS of a resuscitation team in real time is challenging due to the emergency department (ED) environment. [7] One study showed that traditional review only detected 20% of errors that were seen in video review [8], highlighting the opportunity to enable forensic review of team performance. Clinical work must be examined in its natural setting to allow for inclusion of the nuances of real-life not accounted for in simulation. Introducing video review into the ED allows for critical review to gain insight from others. [9].

Video review in medicine is established in many contexts, including simulations and surgery. [10] Although not widely used in the ED yet, some hospitals use it to assess NTS to support teaching and quality improvement. [11] There is currently no consensus on assessment of NTS using video review in the ED. [12].

### Aims

- To provide an overview of tools used to assess NTS in resuscitation teams within the ED using video review.
- To explore to evidence for the validity and usability of the tools.

## Methods

**Fig. 1:**
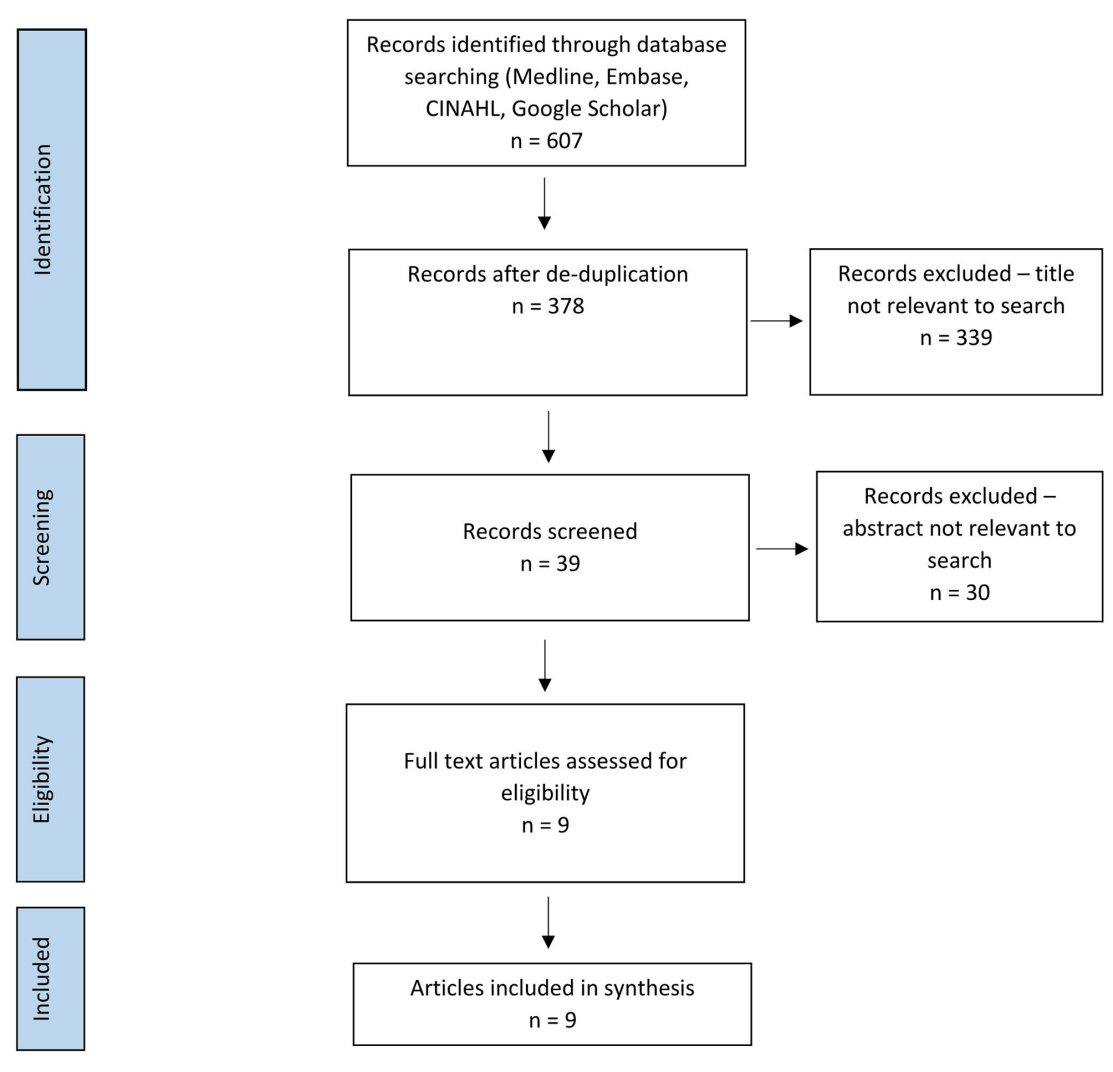
PRISMA diagram for NTS assessment tools

This review is registered with PROSPERO (Ref No: CRD42022306129). Peer-reviewed studies were identified using electronic databases Medline, Embase and CINAHL. A grey literature search was completed using Google Scholar. A manual search of the reference list of relevant articles was conducted. The PRISMA diagram for review of NTS assessment tools is shown in Fig. 1. [13] The search strategy is further detailed in the supplementary material.

The inclusion and exclusion criteria were informed by the authors’ experiences and familiarity with existing literature. We sought papers available in English and published between January 1995 and September 2023, which studied resuscitation teams within the ED. Terms including other descriptors were included (e.g., trauma teams, resus team). Papers must also describe a tool used to assess at least one component of NTS where video review was utilised in a real clinical setting. Papers exclusively assessing simulation were excluded. Papers exclusively assessing resuscitation of paediatric patients were excluded due to the challenges presented by paediatric patients lying outside the scope of this paper.

The search criteria were checked by two independent reviewers. Papers for potential inclusion were checked for relevance by title and abstract (see Fig. 1 for PRISMA flowchart process). Relevant papers were retained for full review. Two papers did not have a full paper associated with their abstract, and one was not available in English. In the initial search, no papers required adjudication by the senior author as agreement between reviewers was achieved.

In the final analysis, three structured tools were found (T-NOTECHS, CALM and TTCA-24) and one tool assessing communication (Communication tool). Studies were analysed over three main domains: method of development, applicability and context use of tool, and evidence of validity. Data were collected and synthesised by one author and checked by another.

Risk of bias was considered throughout data analysis and interpertation. Potential bias includes study selection bias, language bias and anchoring bias. One author published a review in situational awareness, [14] a key component of NTS, which may lead to familiarity bias. Mitigations for these risks include review by three authors, use of a systematic search method, repeated re-examinations of papers in a random order, attempting to access pre-published papers from authors and an English translation. The latter were unsucessful as shown in Fig. 1.

All reviewed articles were quality assessed using the Mixed Methods Appraisal Tool (MMAT) Version 2018 [15] by two authors. Discrepancies were discussed until agreement was reached. MMAT is a “critical appraisal tool designed for appraisal… of systematic mixed studies reviews”. Its validity and reliability meet accepted standards and it was pilot tested for reliability in systematic reviews. [16, 17].

Lack of homogeneity in design, definition, and study populations precluded the use of meta-analytic techniques. Findings were tabulated and summarised by detailed narrative analysis in accordance with the PRISMA checklist. [18].

## Results

The screening process is shown in Fig. 1 as per PRISMA guidance. There were 378 discrete studies screened, 339 were eliminated based on title relevance and 28 were eliminated on abstract relevance. A total of 12 studies were assessed for eligibility and 9 were included in the final study.

The summary of characteristics of studies is shown in Table 1. Six observational studies, two retrospective reviews, and one randomised controlled pilot study were included. The trials were conducted in the Netherlands [19, 20], USA [21–24, 27]^,^ Lithuania [25] and Canada [26]. Van Maarseveen et al [20] did not declare duration of time over which data was collected. The other studies were conducted over a mean of 6.94 months (range 2–24).

**Table 1:** Characteristics and findings of included studies

Paper, Year, Country	Type of Study	Length of Study (months)	Patient population	Sample Size	Tool used	Methodology
Bergs et al. [19], 2005, Netherlands	Observational	4	Patients RTS* <12 assessed by major trauma team, > 12 assessed by minor trauma team	193	Communication tool	Resuscitations consecutively enrolled. Information transfer evaluated for all ABCDE’s†. Observer trained and first 30 videos excluded.
Van Maarseveen et al. [20], 2020, Netherlands	Retrospective review	*None stated*	Patients whose condition is severe enough to activate trauma team	18	T-NOTECHS	Pre-power calculation determined 18 videos needed for analysis using 3 reviewers and T-NOTECHS. Investigated interrater reliability.
Dumas et al. [21], 2020, USA	Observational	24	Patients who underwent an emergency department thoracotomy (EDT)	61	T-NOTECHS	Used T-NOTECHS to evaluate NTS. Modified to 3-point Likert scale.
Nagaraj et al. [22], 2021, USA	Retrospective review	3	Patients with level 1 trauma or an injury severity score (ISS) > 15	99	T-NOTECHS	Compared standard of handover from paramedics to T-NOTECHS score.
Steinemann et al. [23], 2012, USA	Observational	6.5	Blunt, multisystem trauma in nonpregnant patients > 6 years	69	T-NOTECHS	Developed T-NOTECHS. Interrater reliability and correlation assessed.
Kava et al. [24], 2019, USA	Prospective randomised controlled pilot study	2	Patients triaged as a resuscitation	20	CALM	Postgraduate year 2 and 3 residents acted as team leader for resuscitation. Intervention group reviewed performance before 2nd attempt. Used CALM tool.
Aukstakalnis et al. [25], 2020, Lithuania	Observational	8	All emergency department resuscitation patients > 18 years	143	T-NOTECHS	Audio / video review process to evaluate technical and non-technical skills performance. Used T-NOTECHS.
Bhangu et al. [26], 2022, Canada	Observational	2	Patients for whom a trauma code was activated	55	T-NOTECHS	2 independent reviewers used T-NOTECHS with 5 point Likert scale to assess NTS using video review.
DeMoor et al. [27], 2017, USA	Observational	6	Patients recorded as trauma resuscitations (1:1 ratio of stable:unstable patients)	70	TTCA-24	Compared TTCA-24 to T-NOTECHS and TEAM tool scores for same videos to assess concurrent validity

*RTS = Revised Trauma Score; †ABCDE’s = Airway, Breathing, Circulation, Disability, Exposure

There was heterogeneity between studies in relation to patient groups, outcome measures and methodology. All studies were single centre studies due to methodology. The key findings of the studies are highlighted in Table 2.

**Table 2:** Main findings of included studies

Paper, Year	Main Aim	Main Outcome Measure	Reviewers, interrater reliability	Key findings	Limitations	Conclusions
Bergs et al., 2005 [19]	Document information transfer during multi-disciplinary team resuscitation	Presence of audible communication (16–74%)	1, n/a	Better communication in major trauma team than minor trauma team. Better communication during exposure of severely injured patient (p = 0.06)	No formal taxonomy used	Lack of feedback in current training system. Communication is sub-optimal. Other industries have better guidelines
Van Maarseveen et al., 2020 [20]	Assess reliability of T-NOTECHS	Interrater reliability using ICC*	3, ICC = 0.94 (0.87–0.98)	Used T-NOTECHS. Average ICC excellent across all 5 domains	Unable to assess intra-observer variability. Used non-experts to assess.	High ICC in all domains. T-NOTECHS is reliable, especially with mean of 3 assessors
Dumas et al., 2020 [21]	Evaluate association between T-NOTECHS scores and ROSC in patients undergoing an EDT	Return of Spontaneous Circulation (ROSC)	3, ICC not calculated	Association between T-NOTECHS scores and ROSC did not reach statistical significance. Assessment and decision-making high scores were 5.3x more likely to lead to ROSC	Small sample size. Did not calculate ICC. Knowledge of outcome may bias evaluation. Not all treatment specific outcome captured.	Demographic and injury data not associated with ROSC in univariate analysis
Nagaraj et al., 2021 [22]	Characterise EMS handoff experience and effects of non-technical performance	T-NOTECHS, median 10/15 (3-point Likert scale)	1, n/a	Significant difference in score based on experience of team leader. There is a relationship between quality of handover and T-NOTECHS score.	Cessation of videos cause some videos to be excluded. Inability to adjust audio based on noise level.	No significant difference on whether team leader was present, only level of experience
Steinemann et al., 2012 [23]	Develop a tool on 5 essential behavioural domains	T-NOTECHS, mean 16.3–17.7/25 (5-point Likert scale)	2, ICC = 0.48	Significant correlation between T-NOTECHS ratings and standard outcomes such as mortality and length of stay, as well as higher T-NOTECHS correlating with faster resuscitation	Moderate inter-rater reliability and inexperienced raters	Preparedness and resilience associated with situational awareness enabled teams to cope
Kava et al., 2019 [24]	Assess utility of video review compared with self-reflection alone	Assessing gain scores in residents with and without self-reflection using video review between resuscitations acting as team leader.	2, weighted Kappa = 0.45	Positive effect of incorporating video review feedback into leadership training for EM	Convenience sampling. Variability in time to compete self-reflection	Potential use is direct coaching from mentor while watching together
Aukstakalnis et al., 2020 [25]	Create framework for trauma team performance analysis in ED	T-NOTECHS, mean 11.9/25 (5-point Likert scale used)	1, n/a	ISS† > 15 in 16% of cases. 5.6% trauma patients died. Mean rate of completion of primary survey was 68.5%. Poor assessment and decision making. Low levels of ATLS guideline compliance	One video recording reviewer – fine for timings, bias for T-NOTECHS. Poor sound quality during severely injured resuscitation due to overcrowding and shouting	Larger variability supports loss of standardisation. Performs worse when compared to other studies – lack of briefing, room crowding and poor non-technical skills. Trauma team unfamiliar with each other
Bhangu et al., 2022 [26]	Determine whether T-NOTECHS can be used to identify communication gaps in the trauma bay	T-NOTECHS, median 22/25 (5-point Likert scale used)	2, ICC = 0.52	ISS > 16 in 37% of cases. Communication and interaction significantly lower median score (p < 0.0001) when compared to other domains. More completed closed loop communications in severe cases.	Moderate inter-rater reliability. Limitation on team members due to COVID-19 protocols.	Video review provides an opportunity to identify areas for improvement, such as communication, which can be identified by the T-NOTECHS tool. Video review can further be used to assess callouts and closed loop communication.
DeMoor et al. [27]	Demonstrate TTCA-24 is a valid and reliable measurement of communication	TTCA-24	No. of reviewers not stated, ICC = 0.78 (unstable) and 0.87 (stable)	Spearman rank correlation coefficient between TTCA-24 and T-NOTECHS was r = 0.261. No correlation with TEAM score. High interrater reliability of TTCA-24.	TTCA-24 places heavy focus on communication and lacks evaluation of other aspects of NTS	TTCA-24 provides more in depth analysis to communication as a NTS but forgoes a broader impression of NTS as a whole.

*ICC = inter-class correlation coefficient; †ISS = injury severity score

**Fig. 2:**
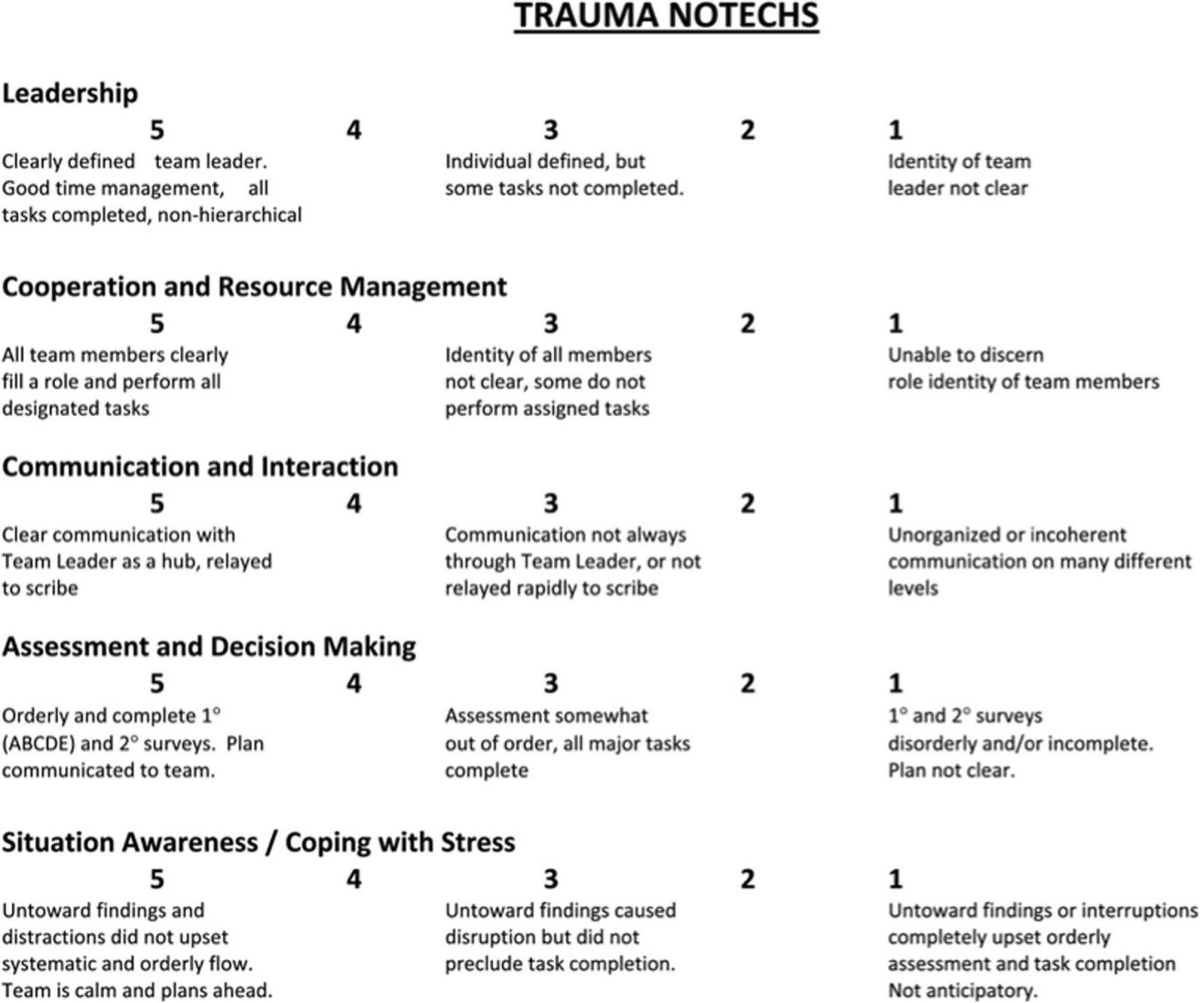
T-NOTECHS tool [23]

**Fig. 3:**
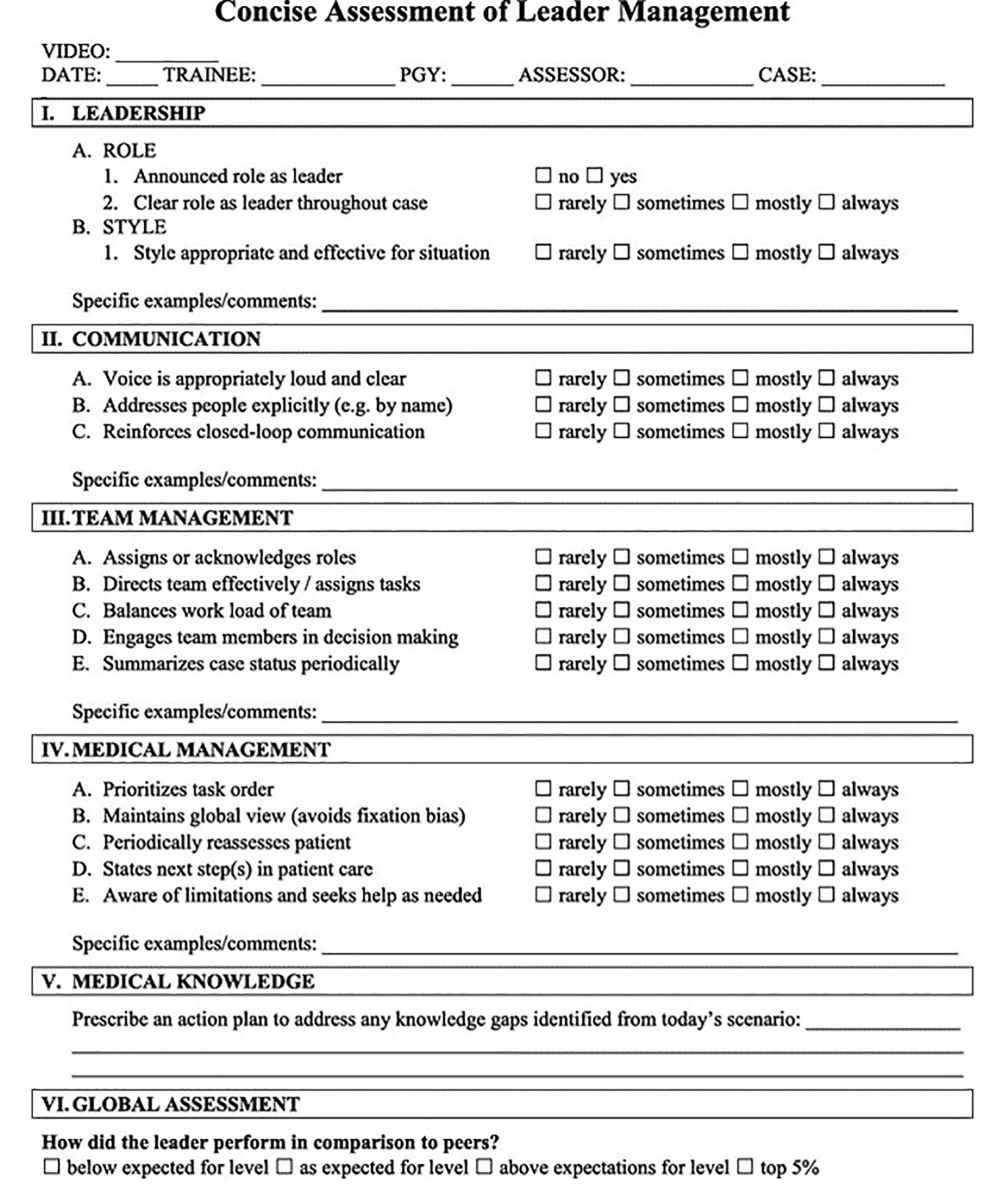
CALM tool

**Fig. 4:**
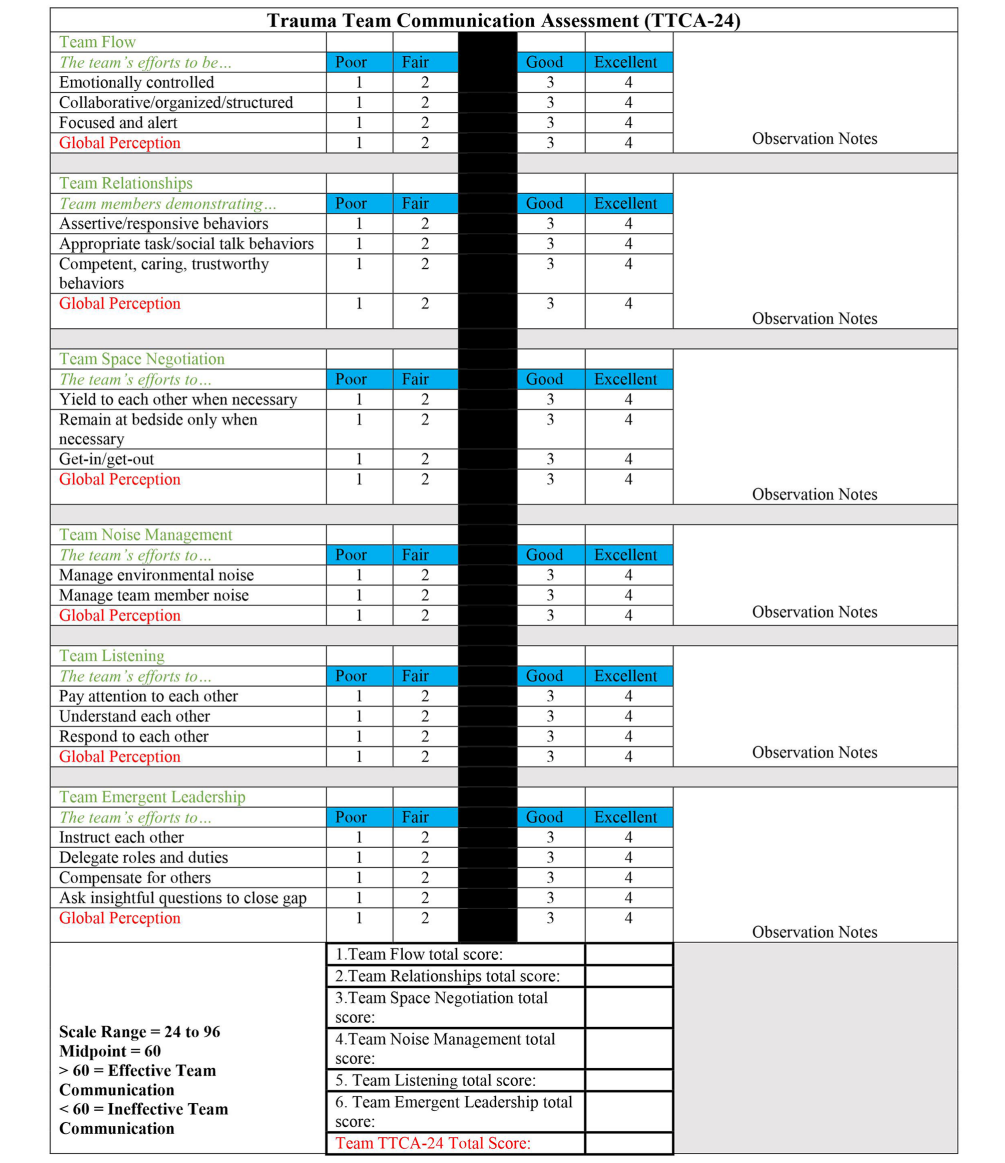
Similarities and differences between the tools identified

Four unique tools for assessing NTS in this setting were identified. The Communication tool was used to assess whether communication was audible or absent [19]. Three structured tools were identified: the Trauma Non-Technical Skills Assessment Tool (T-NOTECHS), the Concise Assessment of Leader Management (CALM) tool and the Trauma Team Communication Assessment (TTCA-24). The components of T-NOTECHS, CALM and TTCA-24 are shown in Figs. 2, 3 and 4 respectively [19, 23, 27].

T-NOTECHS, CALM and TTCA-24 measure NTS, however, they score components differently. T-NOTECHS splits NTS into 5 distinct categories with a 5-point Likert scale for each heading, whereas CALM and TTCA-24 utilise a 4-point Likert scale for each individual element under its headings. They all assess leadership, communication, and general team performance; however, they adopt individual approaches.

The Mixed Methods Appraisal Tool (MMAT) was used to assess quality of papers; however, this was limited by the variability in provision of evidence [15]. The highest quality papers by MMAT standards was Bergs et al [19] and DeMoor et al [27] with 100% quality review. The T-NOTECHS papers scored a mean of 87.5% (range 75–100%) [20–23, 25, 26]. Kava et al [24] scored 80%, however, there were five sections to score this paper due to its methodology compared to four sections for the others.

Reliability was assessed within studies using inter-class correlation coefficient (ICC). Rater reliability represents the extent to which the data collected in the study correctly represents the variables measured. [28] The T-NOTECHS papers which used ICC were Steinemann et al [23] who had an ICC score of 0.48 for real-life resuscitations and van Maarseveen [20] et al. had an ICC of 0.94 (0.87–0.98). Steinemann et al [23] report poor reliability and van Maarseveen et al [20] report excellent reliability. [29] Bhangu et al [26] had an ICC score of 0.52 but did not comment on the reliability.

The CALM paper, Kava et al [24], used weighted Kappa between two experts to ensure agreement and it was 0.45 (CI 0.35–0.56, *p* < 0.0001). This is a weak level of agreement. [28] Both ICC and weighted Kappa can be used to assess inter-rater reliability. The other papers did not demonstrate assessment of reliability.

The TTCA-24 paper, DeMoor et al [27], assessed the ICC in both stable and unstable patient activations and reported 0.87 and 0.78, respectively, which demonstrates excellent reliability. [29].

T-NOTECHS is suggested to be a more reliable assessment of NTS than CALM, as inter-rater reliability is higher across the studies that assess it. Both T-NOTECHS and the CALM tool are previously validated in the literature. [23, 30]. The TTCA-24 demonstrates excellent reliability however, there has only been one study published so far in this context by the author of the tool. The T-NOTECHS reliability is more variable across studies, however, has been better studied.

## Discussion

The first tool for assessing NTS in healthcare was developed by Gaba et al [31] in 1998. This was an adaption of an instrument called NOTECHS where performance was assessed using video recordings from simulated resuscitations [32] in the context of anaesthetic practice. They found high levels of team variability and concluded that the rating system needs refinement before effectively assessing clinical competence. [31] A number of tools have been validated in clinical context, and although Gaba et al [31] is a different context than this review, it demonstrates validity of using video review to assess NTS in simulated resuscitations.

Bergs et al [19] used the Communication tool to assess presence of audible information transfer from physcian to team members. The tool focused on a single element, communication, an important NTS and function of leadership and teamwork. They assessed 204 recordings in a single centre. There was a trend towards better communication during care of the severely injured patient (*p* = 0.06). Some information may not have been picked up due to background noise, a confounder which is not corrected for. Bergs et al [19] concluded communication was sub-optimal.

T-NOTECHS was adapted from NOTECHS, a tool previously used in aviation [5]. which had to be validated for clinical application using several steps. [33] Firstly, a draft tool must be developed. This was done for use in the trauma context by Steinemann et al. [23]. Then, a tool must be adapted based on findings of pilot data. Adaptions of T-NOTECHS between papers in this review are the variation in the number of points in the Likert scale used. Five papers used the original 5-point Likert scale. [20, 23, 25–27] The other two papers [21, 22] utilised the same headings, but reduced the respective scales to a 3-point Likert scale. No study has been identified to validate this contraction. The 5-point scale is more accepted in practice due to increased reliability and validity, alongside its ability to identify extreme attitudes. [34] One paper argued that 3-point Likert scales introduce rounding error but they are quicker to complete which increases the usability. [35] Finally, a tool becomes validated when “researcher has come to the opinion that the instrument measures what it was supposed to measure”. [20, 33] In the context of measuring NTS in a trauma setting, the application of T-NOTECHS by more studies shows that authors of further studies agree with the findings of Steinemann et al., [23] and applied the tool to their own studies. [20–23, 25] The T-NOTECHS scale is shown in Fig. 2.

The CALM tool was developed by Nadkarni et al [30] in 2018 and validated in paediatric simulations to assess team leader performance. It was applied to adult real-life resuscitations by Kava et al [24] to assess individual resident performance as team leader. The CALM tool is shown in Fig. 3. It assessed 15 NTS components which is more than the 5 components assessed in T-NOTECHS, providing a greater scope of assessment. T-NOTECHS may be able to give a greater insight into smaller range of NTS assessed.

The TTCA-24 tool was designed by DeMoor et al [27] as they commented on the use of T-NOTECHS and the Team Emergency Assessment Measure (TEAM) developed by Cooper et al[38]. The senior author felt that these tools lacked scope to adequately assess communication as a NTS so developed the TTCA-24 tool to be used live or during video review. DeMoor et al. assessed concurrent validity between TTCA-24 and T-NOTECHS and TTCA-24 and TEAM. The Spearman rank correlation coefficient between TTCA-24 and T-NOTECHS is r = 0.261, demonstrating positive correlation that was statistically significant (p = 0.029). There was no statistically significant correlation between TTCA-24 and TEAM. As T-NOTECHS contains a distinct communication category, it is understandable how these tools would be correlated.

The T-NOTECHS, CALM and TTCA-24 tools both assess leadership, communication and team managment. T-NOTECHS emphasises decision making and situational awareness, CALM focuses on medical management and knowledge and TTCA-24 focuses on team communication. These are not distinct categories and demonstrate overlap in some areas. T-NOTECHS recognises the response to “untoward findings”, a useful inclusion that helps to validate its use in real-life resuscitations, as this is common in the ED. [36] T-NOTECHS and TTCA-24 are designed to assess team performance, whereas CALM is better suited to assessing individual performance.

All tools demonstrate a high level of usability. T-NOTECHS provides an explanation for the lowest, highest and middle score to guide the user. CALM uses a simple scoring system which enables the user to assess the frequency at which each NTS is exhibited. T-NOTECHS is potentially easier to complete as limited number of components to rate. When paired with video review, reviewers can pause or rewind the video for a more accurate assessment of NTS. [23] The TTCA-24 tool was designed for interprofessional use and comes with a codebook. The high inter-rater reliability suggests that the raters utilised the tool in the same way. The inter-rater reliability of TTCA-24 is highest of the three, however, it has both TTCA-24 and CALM have only been utilised in this context in one paper so more research is needed. T-NOTECHS has more variable ICC across studies, so more research would be beneficial in getting a truer picture of ICC across a larger sample size. [20–27].

Higham et al [1] evaluated tools used for assessment of NTS in healthcare. Due to broader inclusion criteria, this study identified 76 distinct tools, including T-NOTECHS, for assessment of NTS. They noted a large amount of variation between methodology of design of tools, extent of their validity and usability. This was also evident in the comparison of our three assessed tools. They suggest that there is a “need for rationalisation and standardisation in the way we assess non-technical skills in healthcare”. This study was published in 2019 and included Steinemann et al. [23], and 6 out of 7 of the studies we reported that used T-NOTECHS were published later. The inclusion of the newer studies in our review furthers the research into the standardisation of assessment of NTS.

Bhangu et al [37] also published a scoping review in 2022 evaluating tools used to assess NTS in both real world and simulated settings. They identified the T-NOTECHS and TEAM tool as the most reliable for use in this context. The TEAM tool was used in studies utilising simulation which means they do not fit the inclusion criteria for this review. This tool was adapted from a paper by Cooper et al. in 2010 [38] and further validated in 2016 [39] in both simulated and real-life settings, without video review. No studies included in this review utilised the TEAM tool.

The aim of this review was to provide an overview of tools used to assess NTS in resucitation teams within the ED using video review and to explore the evidence for the validity and usability of the tools. This review has answered the stated aims despite having a limited number of papers included. We found T-NOTECHS to be the most valid tool and has been shown to be a reliable tool to assess NTS during resuscitation in the emergency deparment using video review. The TTCA-24 tool showed early signs of good reliability but will need to be further validated. The TTCA-24 provides more insight into communication as a NTS than T-NOTECHS, but when assessing NTS more holistically, T-NOTECHS demonstrates usability, reliability and validity. The authors are aware of the difficulty of excluding bias and can hope that the techniques utilised minimised bias.

Due to the heterogeneity of studies, there was limited application of statistical approaches to compare tools. A similar review identifies a need to benchmark outcomes between studies, thus enabling a potential future meta-analysis. [40] The findings of our review provide more clarity on the use of T-NOTECHS as a standardised tool which would enable use of video review as a tool in education and quality improvement. [41] One study translated T-NOTECHS into Finnish to assess translatability and validity and found that it can still be used to assess efficacy of trauma team resuscitations. This study used simulated trauma resuscitations, which was an exclusion criteria for our review. [34].

Steinemann et al [23] also assessed use of T-NOTECHS in the context of simulated resuscitations using video review. Rater agreement was higher in simulated resuscitations than in real-life resuscitations (ICC = 0.71). There was a significant correlation found between the number of completed resuscitation tasks (*r* = 0.50, *P* = < 0.01) and faster time to completion of the 3 common resuscitation tasks (*r*=-0.38, *P* < 0.05). [23] Simulated resuscitations are a useful tool to assess NTS of staff as there are less ethical considerations when filming patients. However, the nature of the simulated environment does not provide assessors with a true picture of how teams would perform in a real life clinical setting, hence the exclusion from our review.

This review highlights the tools used in this setting and recommends use of T-NOTECHS to assess NTS in resuscitation teams within the ED using video review. In terms of future study, using T-NOTECHS with larger sample sizes, such is in a multi-centre study may greatly establish utility of this tool. TTCA-24 may have uses in departments where communication is identified as a weakness by the use of T-NOTECHS or other means. Both tools can be used to identify areas where further clinician education is indicated. Furthermore, there is scope to formally compare NTS with TS using video review within the ED.

### Limitations

One of the limitations of this review is the small sample size. There is a breadth of tools available that assess NTS across all domains of healthcare, however, use of video review in the ED is a growing field and excluding studies without video review reduced the number available. Due to the infrastructure and resource demands to review video creation and validation of a new tool and demonstrating generalisability will be challenging. Use of tools developed and validated in the simulation context requires demonstration of their utility in real-world clinial care.

Many institutions lack audio-visual recording access due to finanacial and ethical restraints, therefore there is limited generalisability for these findings. Researchers may be faced with a reluctance to be filmed due to privacy concerns from staff regarding patients and themselves. There should be strict measures in place to ensure recordings are only accessed by appropriate personnel to ensure privacy and security.

## Conclusion

The aim of this review was to provide overview of tools used to assess NTS in resuscitation teams within the ED using video review and to explore the evidence for the validity and usability of the tools. T-NOTECHS was first validated in Steinemann et al [23] and therefore was the tool of choice for the majority of future papers assessing NTS in the ED using video review. This review found T-NOTECHS to be valid and reliable. The conclusion that T-NOTECHS is the best tool of those used in this context is suggested, but not able to be proven fully due to small sample sizes.

Acknowledgements:

### Electronic supplementary material

Below is the link to the electronic supplementary material.

Supplementary Material 1

## Data Availability

All of the material is owned by the authors and no permissions are required. For access to raw data analysed, contact Emily G Alexander at 237165a@student.gla.ac.uk.
