# Supplementary material for: Evaluation of video review tools for assessing non-technical skills in emergency department resuscitation teams: a systematic review
Source: BMC Emerg Med. 2023 Nov 29;23:141. doi: 10.1186/s12873-023-00895-7 (PMC10687788; doi:10.1186/s12873-023-00895-7)
Supplement: Supplementary file 1 — Supplementary Material 1 [file 12873_2023_895_MOESM1_ESM.docx]

# Search Strategy

Aim: to provide an overview of tools used to assess NTS in resuscitation teams within the ED using video review

Initial limited search of a selection of relevant databases (Medline and Embase), followed by an analysis of text words contained in the title and abstract, and of the index terms used to describe the article. The original key words used were suggested by the senior author who has familiarity and experience with this area of research. Similar and relevant papers were searched for key words. This method was then used to identify a full list of key words to be used in the search.

A second search using all identified keywords and index terms is the undertaken across all databases. Phrase searching was also used.

Boolean operators were applied to broaden the search on distinct topics and also capture articles where all concepts appear. No search limits were used. Upon review, citation of relevant articles were searched to ensure no articles were missed. Only papers fully published in English will be considered for review.

The search strategy was checked independently by two authors (E.A. and C.M.). Any papers where agreement was not reached were checked by the senior author (D.J.L.). Agreement on inclusion of papers was reached by all three authors.

Medline, CINAHL, Embase and Google Scholar

Search date: 3^rd^ September 2023

1. trauma video review.ti,ab,kw.
2. video recording.ti,ab,kw.
3. video review.ti,ab,kw.
4. video analysis.ti,ab,kw.
5. video-based analysis.ti,ab,kw.
6. video evaluation.ti,ab,kw.
7. video cameras.ti,ab,kw.
8. trauma video.ti,ab,kw.
9. video assessment.ti,ab,kw.
10. video based analysis.ti,ab,kw.
11. trauma resuscitation.ti,ab,kw.
12. resuscitation.ti,ab,kw.
13. resuscitation team.ti,ab,kw.
14. team performance.ti,ab,kw.
15. leadership.ti,ab,kw.
16. multidisciplinary team.ti,ab,kw.
17. multidisciplinary cooperation.ti,ab,kw.
18. patient care team.ti,ab,kw.
19. clinical competence.ti,ab,kw.
20. cooperative.ti,ab,kw.
21. group process.ti,ab,kw.
22. emergency department.ti,ab,kw.
23. emergency room.ti,ab,kw.
24. accident.mp. and emergency.ti,ab,kw. [mp=ti, bt, ab, ot, nm, hw, fx, kf, ox, px, rx, ui, sy, ux, mx, tn, dm, mf, dv, dq]
25. A&E.ti,ab,kw.
26. ED.ti,ab,kw.
27. ER.ti,ab,kw.
28. resuscitation room.ti,ab,kw.
29. resus.ti,ab,kw.
30. resuscitation.ti,ab,kw.
31. trauma centre.ti,ab,kw.
32. trauma unit.ti,ab,kw.
33. emergency.ti,ab,kw.
34. trauma.ti,ab,kw.
35. trauma NOTECHS.ti,ab,kw.
36. NOTECHS.ti,ab,kw.
37. non technical skills.ti,ab,kw.
38. non-technical skills.ti,ab,kw.
39. clinical competence.ti,ab,kw.
40. clinical competency.ti,ab,kw.
41. interprofessional relationships.ti,ab,kw.
42. trauma evaluation.ti,ab,kw.
43. task performance analysis.ti,ab,kw.
44. 1 or 2 or 3 or 4 or 5 or 6 or 7 or 8 or 9 or 10
45. 22 or 23 or 24 or 25 or 26 or 27 or 28 or 29 or 30 or 31 or 32 or 33 or 34
46. 11 or 12 or 13 or 14 or 15 or 16 or 17 or 18 or 19 or 20 or 21 or 35 or 36 or 37 or 38 or 39 or 40 or 41 or 42 or 43
47. 44 and 45 and 46
48. remove duplicates from 47
